# Management of Pulmonary Toxicities Associated with Systemic Therapy in Non Small Cell Lung Cancer

**DOI:** 10.1007/s11864-024-01257-6

**Published:** 2024-09-20

**Authors:** Marko Velimirovic, Matthew Brignola, Emily Chheng, Michael Smith, Khaled A. Hassan

**Affiliations:** 1 Department of Thoracic Oncology, Cleveland Clinic, Taussig Cancer Institute, 10201 Carnegie Ave, Cleveland, OH 44106 USA; 2 Department of Pharmacy, Cleveland Clinic, Taussig Cancer Institute, Cleveland, OH USA; 3 Department of Pulmonary Medicine and Critical Care Medicine, Cleveland Clinic, Respiratory Institute, Cleveland, OH USA

**Keywords:** Pneumonitis, Drug-induced, Lung cancer, Immunotherapy, EGFR inhibitors

## Abstract

Drug-induced pneumonitis is a common adverse event that may occur during lung cancer systemic therapy. The incidence/prevalence of this side effect has increased due to recent extensive use of immunotherapy. Although pneumonitis prevalence is increased with the use of immune checkpoint inhibitors, it is also associated with chemotherapy and targeted therapy. Pneumonitis can occur early after drug exposure or present after several cycles of treatment. Its severity can range from insidious to fulminant, leading to hospitalization. In most cases, the diagnosis is made based on medical history, temporal correlation with use of lung cancer systemic therapy, and computed tomography (CT) findings. In the majority of cases, stopping the offending drug and use of corticosteroids is the sufficient treatment; however, patients with more severe forms of pneumonitis require additional immunosuppressive agents. In this review, we address pneumonitis caused by chemotherapy, antibody–drug conjugates, targeted therapy, or immunotherapy, and provide a detailed management approach.

## Introduction

Lung cancer has been the leading cause of cancer related death in the USA. Overall survival has been improving over the years, mainly due to the advancement of new systemic treatment options. Cytotoxic chemotherapy had been the cornerstone of treatment of advanced lung cancer for decades, until the incorporation of targeted therapy in the 2000's. EGFR-targeted agents against classical mutations in exon 21 and exon 19 deletion introduced a new treatment approach for lung cancer. The discovery of specific driver mutations led to the development of targeted drugs that have better response rates and fewer side effects. The approval of EGFR-targeted therapies paved the way for several drugs targeting at least eight different signaling pathways. As the field was dissecting lung cancer into different subgroups to deliver unique specialized therapies, success stories in targeting the tumor environment and exploiting the strength of the immune system were emerging. Checkpoint inhibitor immunotherapy has transformed the field and is now utilized in both early and advanced setting. With the positive impact of immunotherapy on lung cancer outcomes, identifying and treating side effects is paramount. This review article addresses pneumonitis that occurs as a complication of different systemic therapeutic options. Most of the current data regarding pneumonitis due to lung cancer systemic therapy comes from the use of immunotherapy. Thus, immune checkpoint inhibitor-induced pneumonitis (ICI-P) is used as the paradigm for pneumonitis caused by other agents.

## Diagnosis

ICI-P is a diagnosis of exclusion, however, due to significant morbidity and potential prolonged treatment interruptions, any suspicion of ICI-P requires prompt and thorough diagnostic evaluation. Grading of ICI-P ranges from asymptomatic to severe, necessitating hospitalization and mechanical ventilation [1] (Table 1). Asymptomatic patients (grade 1) are typically diagnosed with ICI-P based on radiographic changes discovered on their regularly scheduled restaging scans. Grade 2–4 ICI-Ps are characterized by new or worsening dyspnea, dry cough, chest pain, and/or fever, which should prompt pulmonary function testing and lung imaging with dedicated chest CT outside of the restaging imaging cycle.

**Table 1:** Management of Immune-Related Adverse Events in Patients Treated With Immune Checkpoint Inhibitor Therapy: ASCO Guideline Update 2021

Workup and evaluation Should include the following: Pulse oximetry and CT chest preferably with contrast if concerned for other etiologies such as pulmonary embolus For G2 or higher, may include the following infectious workup: nasal swab, sputum culture, and sensitivity, blood culture and sensitivity, urine culture, and sensitivity COVID-19 evaluation- per institutional guidelines where relevant	
Grading	Management
G1: Asymptomatic; confined to one lobe of the lung or < 25% of lung parenchyma; clinical or diagnostic observations only	Hold ICPi or proceed with close monitoring Monitor patients weekly with history and physical examination, pulse oximetry; may also offer chest imaging (CXR, CT) if uncertain diagnosis and/or to follow progress Repeat chest imaging in 3–4 weeks or sooner if patient becomes symptomatic In patients who have had baseline testing, may offer a repeat spirometry or DLCO in 3–4 weeks May resume ICPi with radiographic evidence of improvement or resolution if held. If no improvement, should treat as G2
G2: Symptomatic; Involves more than one lobe of the lung or 25%-50% of lung parenchyma; medical Intervention Indicated; limiting instrumental ADL	Hold ICPi until clinical improvement to ≤ Gl Prednisone 1–2 mg/kg/d and taper over 4–6 weeks Consider bronchoscopy with BAL ± transbronchial biopsy Consider empiric antibiotics if infection remains in the differential diagnosis after workup Monitor at least once per week with history and physical examination, pulse oximetry, consider radiologic imaging; if no clinical improvement after 48–72 h of prednisone, treat as grade 3 Pulmonary and infectious disease consults if necessary
G3: Severe symptoms; Hospitalization required: Involves all lung lobes or > 50% of lung parenchyma; limiting self-care ADL; oxygen indicated G4: Life-threatening respiratory compromise; urgent intervention indicated (intubation)	Permanently discontinue ICPi Empiric antibiotics may be considered Methylprednisolone IV 1–2 mg/kg/d If no improvement after 48 h, may add immunosuppressive agent. Options include infliximab or mycophenolate mofetil IV or IVIG or cyclophosphamide (See Table A2 for dosing). Taper corticosteroids over 4–6 weeks Pulmonary and infectious disease consults if necessary May consider bronchoscopy with BAL ± transbronchial biopsy if patient can tolerate

The most common radiographic patterns seen in ICI-P are organizing pneumonia (OP), hypersensitivity pneumonitis (HP), and nonspecific interstitial pneumonia (NSIP). These CT patterns often correlate with grades of pneumonitis toxicity, according to the Common Terminology Criteria for Adverse Events (CTCAE). The highest CTCAE grades and worst prognoses are observed in diffuse alveolar damage (DAD) and OP, while the lowest grades are seen in NSIP and HP patterns [2, 3] (Table 1). Radiographic changes can be confined to a single lobe or be diffuse, and the extent of the affected lung parenchyma is one of the criteria for ICI-P grading (Table 1). Experienced radiologists are often able to diagnose ICI-P with reasonable accuracy, however bronchoscopy for bronchoalveolar fluid sampling and lung biopsy may be needed in some cases.

The differential diagnosis of ICI-P includes infectious pneumonias, adverse reactions to other concomitantly used drugs, such as antimicrobials or antiarrhythmics, radiation and/or recall pneumonitis, de novo interstitial lung disease (ILD), and pulmonary spread of the underlying malignancy. Bronchoscopy is largely used to exclude underlying infection and progression of disease as there are no pathognomonic findings in either the bronchoalveolar lavage (BAL) or lung biopsy to reliably diagnose ICI-P. Despite this, there are clues in the BAL that can lead to earlier presumptive diagnosis of ICI-P to begin treatment. Recent data suggests that an increase in the percentage of lymphocytes in the BAL is common in ICI-P, and their presence can aid in diagnosis [4–9]. Less commonly, an increase in the percentage of neutrophils and eosinophils can also be seen, and thus their presence does not exclude ICI-P [5]. There is only sparse data available about the biopsy features of ICI-P; typical findings include cellular interstitial pneumonitis, organizing pneumonia, diffuse alveolar damage, poorly formed granulomas, and eosinophil infiltration [10]. High resolution computerized tomography (HRCT) findings and clinical history typically correlate well with surgical biopsy findings [11]. As such, biopsy is usually not necessary for the diagnosis of ICI-P. Transbronchial lung biopsy increases the risk of complications, specifically pneumothorax and bleeding, and thus should be reserved for cases where suspicion for an alternative diagnosis is high and the biopsy results are expected to change management.

## Chemotherapy

### Incidence/Risk Factors

Among the various chemotherapy drugs used in NSCLC, pneumonitis is mainly seen in Taxanes. Docetaxel and paclitaxel (conventional and albumin-bound), are common antimicrotubule inhibitors used in the treatment of lung cancer. While typical side effects such as myelosuppression and peripheral neuropathy are classically reported toxicities in primary literature, rates of pneumonitis secondary to taxanes have not been well defined. The rate of all-grade taxane-induced pneumonitis (TIP) is reported to be 4.6%, but can be higher depending upon patient factors [12, 13]. TIP can occur at any point throughout treatment and is primarily seen within the first 12 weeks, with a median onset of 42 days reported by one study examining docetaxel in non-small cell lung cancer (NSCLC) [13, 14].

Several risk factors may increase the incidence of TIP, including concomitant cytotoxic agents and radiation, prior ILD, and dosing frequency of the taxane [13–15]. A dose-finding study using combined docetaxel and gemcitabine in NSCLC halted recruitment early after 23% of patients experienced pulmonary toxicity. The authors concluded that the combination was too toxic for further study [16]. Several randomized trials corroborate this increased risk of pulmonary toxicity when gemcitabine is used in combination with a taxane, as such this combination is not recommended in NSCLC [16–19]. Combining treatment modalities with taxanes and radiation have been shown to increase risk for TIP; however, this may not translate into an increased risk for treatment-related death [20–23]. Typically, patients with ILD are excluded from clinical trials but there is existing literature providing guidance on the use of taxanes in patients with history of ILD. Shukuya et al*.* demonstrated a 27% rate of grade 3 or higher pneumonitis in patients with ILD treated for NSCLC with combination carboplatin-paclitaxel [24]. Several small studies and case reports confirm these findings and recommend guarded use of taxanes in this population [25, 26]. Interestingly, other studies indicate that an increase in TIP risk has a greater association with the frequency of dosing rather than dose amount. Patients receiving weekly doses of taxanes had higher rates of ILD compared to larger doses given on a 3-week dosing schedule [13, 22, 27]). In contrast, albumin-bound paclitaxel demonstrated a low risk of TIP in patients with lung cancer in one study where 95.7% of the patients were free of ILD exacerbation at the prespecified 28-day endpoint [28].

### Management

Management of TIP is dependent upon severity and initial steps should include holding further treatment with the offending taxane [14, 24, 29]. The use of corticosteroids is the mainstay of treatment for patients with TIP who have more moderate to severe respiratory compromise [14, 29, 30]. Dosing strategies can differ depending upon clinical variables including grading, clinical presentation, and patient preference. No formal recommendations for an optimal dose of glucocorticoids have been accepted by national guidelines but intermediate-acting glucocorticoids (prednisone, prednisolone, methylprednisolone) are most commonly used [29, 31]. Dosing strategies vary and can include flat dose, weight-based, or high-dose pulse, all followed by a prolonged tapering strategy [29, 31]. We recommend following IO therapy pneumonitis treatment guidelines. In those patients who do not respond to corticosteroids, risk of mortality greatly increases and may rise to 50% [30]. While treatment in those unresponsive to corticosteroids is not well-established, the use of alternate immunosuppression has been documented in a case report where etanercept was used for successful treatment of refractory TIP [12]. It should be noted, however, that the causative agent has come under scrutiny, and that oxaliplatin could be implicated as the cause of pneumonitis in this case [32]. Caution should be exercised when using alternative immunosuppressing agents for the treatment of taxane pneumonitis, and other potentially causative agents should be sought out. Finally, taxane rechallenge can be considered depending on patient-specific factors and the clinical course [14]. Rechallenge should only be considered in patients with complete clinical and radiographic recovery of TIP; short courses of prophylactic oral steroids in doses of 0.25–0.5 mg/kg prednisone equivalent for approximately 1 week can be considered to prevent recurrence.

## Antibody–Drug Conjugates

### Incidence/Risk Factors

Historically, a variety of human epidermal growth factor receptor 2 (HER2)-targeted agents have been studied in NSCLC and it was only more recently that the use of antibody–drug conjugates (ADCs) has shown meaningful benefit in patients with lung cancers harboring HER2 mutations. Anti-HER2 targeted ADCs, trastuzumab-deruxtecan (T-DXd) and trastuzumab emtansine (T-DM1), were first introduced in the treatment of HER2-overexpressing breast cancers but are a relatively new addition in lung cancer care. The risk for ILD/pneumonitis is an established side effect but the incidence varies depending upon the agent used. HER2 is expressed in normal lung epithelium and increases during acute lung injury, which suggests a plausible basis for the increased risk of pneumonitis or worsening ILD in this patient population [33]. An increased risk for ILD reaching 25% has been seen in patients with lung cancer receiving T-DXd when compared to a pooled rate of 11.4% in patients with other malignancies [34]. The DESTINY-Lung01 trial found a 26% risk for all-grade ILD, which resulted in 2 patients’ deaths when treated with T-DXd (6.4 mg/kg) [35]. DESTINY-Lung02 also studied a lower dosing strategy of 5.4 mg/kg in comparison to 6.4 mg/kg and found decreased risk in ILD [36]. In contrast, the use of T-DM1 is associated with a much lower incidence of ILD [37]. As the use of HER2-targeted ADCs is relatively new, patient-specific risk factors have yet to be fully elucidated. The lung cancer population may be at a higher baseline risk because of their comorbid lung disease.

### Management

Management of HER2-targeted ADC-related pneumonitis is similar to that of TIP. Corticosteroids are the mainstay of treatment and the manufacturers of the most common ADC, T-DXd, have specific management discussed in their trial protocol for pulmonary toxicity [35]. The drug manufacturer has recommended that only patients with grade 1 pneumonitis should be considered for rechallenge. Published literature supports this practice, as well as a multidisciplinary treatment approach to ensure proper care of these patients [38].

## Epidermal Growth Factor Receptor Inhibitors

### Incidence/Risk Factors

Mutations involving the Epidermal Growth Factor Receptor (*EGFR*) gene are one of the more well-known driver mutations in NSCLC. A recent meta-analysis assessing worldwide prevalence of EGFR driver mutations identified a range from 10 to 50% [39]. About 49.1% and 12.8% were identified in the Asian and European populations, respectively. Current EGFR tyrosine kinase inhibitors (EGFR-TKIs) include the first-generation TKIs, erlotinib and gefitinib, the second-generation TKIs, afatinib and dacomitinib, and the third generation TKI, osimertinib. While pulmonary toxicities (including pneumonitis and ILD) associated with EGFR-TKIs are rare, these can be severe (Table 2). The median time to onset of EGFR-TKI-associated ILD is not well-known, however, the landmark phase 3 trial, FLAURA, reported median time to onset of 106 days (range 9 to 425 days) and a case report identified pulmonary toxicity onset as early as five days [40, 41]. Incidences of ILD in randomized controlled trials have ranged from 0 to 4% [40, 42, 43]. Suh et al. conducted a meta-analysis which reported an overall incidence of 1.12% of EGFR-TKI-associated pneumonitis for all grades [44]. Huang et al. assessed the Food and Drug Administration Adverse Event Reporting System (FAERS) database involving the four EGFR-TKIs (gefitinib, erlotinib, afatinib, and osimertinib) from 2004 through 2018. All four agents demonstrated a statistically significant increased risk ILD and pneumonitis [45]. Other studies have also demonstrated a significantly higher incidence of pneumonitis in studies conducted in Japan compared to non-Japanese studies (4.77% vs 0.55% for all grades) [46]. However, this may be due to a higher prevalence of patients with *EGFR* mutations in the Japanese population [47].

**Table 2:** Reported incidences of EGFR-TKI-associated pulmonary toxicities from RCTs and meta-analyses

Author/Type of Study	Tx	AE	Incidence
Hong D et al 2016 Meta-analysis	Gefitinib	High-grade hemoptysis	0.49% (95% CI: 0.24–0.99)
		Pneumonia	2.33% (95% CI: 1.47–3.66)
		Pneumonitis	2.24% (95% CI: 1.34–3.72)
		ILD	1.43% (95% CI: 0.98–2.09)
Suh CH et al 2018 Meta-analysis	EGFRi	Pneumonitis	1.12% (95% CI: 0.79–1.58) - G3 or higher: 0.61% - G5: 0.2% Japanese studies vs non-Japanese origin: 4.77% vs 0.55%, p < 0.001 - G3 or higher: 2.49% vs 0.37%, p < 0.001 - G5: 1% vs 0.18%, p < 0.001
Soria JC et al 2018 Phase 3 Trial (FLAURA)	Osimertinib vs Gefitinib or erlotinib	Pneumonitis	2% vs 1%
		ILD	2% vs 1%
Wu Y et al 2020 Phase 3 Trial (ADAURA)	Osimertinib vs Placebo	ILD	3% vs 0%
Huang J et al 2020 FAERS Database	EGFRi	Pneumonitis	N = 63 ROR 14.83 (95% CI: 11.55–19.04)
		ILD	N = 253 ROR 29.18 (95% CI: 25.67–33.16)
Ohe Y et al 2020 Post-marketing investigation, Japan	Osimertinib	ILD	6.8% (245/3578) - G3 or higher: 2.9% - Mortality: 0.8%
Li X et al 2022 Meta-analysis	ChemoRT vs ChemoRT + EGFRi	Pneumonitis	OR 1.76 (95% CI: 0.98–3.15) p-value 0.06

AE = adverse events, chemoRT = chemoradiotherapy, CI = confidence interval, EGFRi = epidermal growth factor receptor inhibitor, FAERS = Food and Drug Administration Adverse Event Reporting System, G = grade, ILD = interstitial lung disease, N = number of adverse events reports, OR = odds ratio, RCTs = randomized controlled trial, ROR = reporting odds ratio, Tx = treatment

The risk factors and mechanism for EGFR-TKI-associated pulmonary toxicities are not well-understood. Many studies suggest that the male sex, smoking history, pre-existing lung fibrosis, and chronic obstructive pulmonary disease may be contributing factors [48]. It is suggested that EGFR plays a role in lung epithelial repair, thus the inhibition of the EGFR signaling pathway may impair normal response to lung injury [48].

### Management

Treatment of pulmonary toxicities associated with EGFR-TKI therapy is not established and is dependent on grade and severity of symptoms (Table 3). Management includes holding the offending EGFR-TKI agent and the administration of corticosteroids. The consideration of an alternate EGFR-TKI agent or an alternative systemic treatment option should be discussed. A few case reports have demonstrated the successful rechallenge of EGFR-TKIs, both under steroid protection and/or re-introduction of EGFR-TKI at a lower dose followed by titrating to full dose [49–51].

**Table 3:** Management of pulmonary toxicities associated with EGFR-TKIs from case reports

Article	Patient/Tx	Sx	Imaging/Results	Management
Luo C et al 2014 Case report	62 YO male Gefitinib as 2L	Onset of sx: 60d Sx: Dyspnea, dry cough, and fever	Interstitial lung inflammation and bilateral pleural effusion	Treatment: • Held gefitinib • Started broad-spectrum ABX • Started methylprednisolone 1000 mg x 3d Outcome: • Total resolution of ground glass opacities • Restarted gefitinib at reduced dose
Mamesaya N et al. 2017 Case report	38 YO female Osimertinib as 4L	Onset of sx: 31d Sx: SOB and fever	Faint infiltrates in bilateral lung	Treatment: • Held osimertinib
Tachi H et al. 2017 Case report	77 YO female Osimertinib as 4L	Onset of sx: 14d Sx: Hypoxemia and fever	Interlobular septal thickening and bilateral pleural effusion Lung biopsy showed eosinophilic infiltrations	Treatment: • Discontinued osimertinib Outcome: • Clinically improved symptoms
Jobe AL et al 2018 Case report	58 YO female Afatinib as 2L	Onset of sx: 1mo Sx: Oxygen requirement	Increase of ground glass opacities in lung	Treatment: • Held afatinib • Started broad-spectrum ABX • Started methylprednisolone 500 mg daily on hospital day 3 • Discharged with steroid taper (was off of steroids at 1mo follow-up visit) Outcome: • Clinically improved symptoms
Fan M et al 2019 Case report	78 YO male Osimertinib as 2L	Onset of sx: 1mo Sx: Severe cough, difficulty in breathing	Pulmonary space-occupying lesion in lung	Treatment: • Initially recommended to discontinue osimertinib but patient continued treatment • Started methylprednisolone 240 mg daily, broad-spectrum antimicrobial, and mechanical ventilation upon worsening dyspnea Outcome: • Patient died after 2 weeks from multi-organ failure and complications
Hantschel M et al. 2020 Case report	79 YO Osimertinib as 1L	Onset of sx: 13wks Sx: Mild dyspnea	Subpleural and bipulmonary opacities	Treatment: • Continued osimertinib After 3wks, dyspnea worsened: • Required mechanical ventilation • Started prednisolone 500 mg x 3d, followed by 100 mg x 14d Outpatient: • Required steroids over 8 wks. with slow taper • Switched to carboplatin plus gemcitabine
Lu H et al 2020 Case series	61 YO female Osimertinib as 2L	Onset of sx: 3mo Sx: None	Bilateral ground glass opacifications	Treatment: • Continued osimertinib Outcome: • Ground glass opacities improved with no additional management
	57 YO female Osimertinib as 2L	Onset of sx: Within 3wks Sx: Severe dyspnea with AHRF	Extensive bilateral ground glass opacities	Treatment: • Started methylprednisolone 60 mg Q6H x 5d, followed by a 2mo prednisone taper Outpatient: • Switched to systemic chemotherapy – carboplatin and pemetrexed, followed by maintenance pemetrexed Osimertinib re-challenge following progression • Re-introduced osimertinib at reduced dose to every other day initially then daily • Started prednisone 0.5 mg/kg daily, followed by a taper to prednisone 5 mg every other day • Patient did not have any signs of pneumonitis
Mohammed T et al 2021 Case report	71 YO female Osimertinib as 1L	Onset of sx: 1wk Sx: SOB, AHRF	Bibasilar patchy airspace opacities in RLL	Treatment: • Held osimertinib • Started broad-spectrum ABX • Started high-dose steroids (dose and duration not specified) after respiratory failure continued to worsen • Discharged with steroid taper (dose not specified) and osimertinib held Outcome: • Near-total resolution of infiltrates on imaging 6wks after discharge Outpatient: • Restarted osimertinib at a reduced dose then slowly uptitrated to full dose • No further sx reported

ABX = antibiotics, AHRF = acute hypoxic respiratory failure, L = line of treatment, RLL = right lower lobe, SOB = shortness of breath, sx = symptoms, Tx = treatment, YO = years old

## Bispecific Antibody

Another major *EGFR* mutation driving NSCLC is the exon 20 insertion mutation. This mutation alters the kinase binding site and thus prevent the binding of tyrosine kinase inhibitors. This leads to lack of activity of classic EGFR TKI's in patients with this mutation [52]. Amivantamab is a bispecific antibody against mesenchymal epithelial transition factor (MET) and EGFR. Originally Amivantamab was approved as a single agent for patients with metastatic NSCLC who progressed on platinum based chemotherapy [53]. More recently, it was approved in combination with carboplatin and pemetrexed as first-line treatment for patients with metastatic NSCLC with EGFR exon 20 insertion [54]. Pneumonitis occurred in about 3% of patients in both setting either as a single agent or in combination with chemotherapy.

### Management

Similarly to EGFR-TKIs, treatment of pulmonary toxicities associated with Amivantamab includes holding the drug and administration of corticosteroids. Per package insert, the recommendation is to permanently disconitue Amivantmab if pneumonitis is confirmed irrespective of grade.

## Immunotherapy

### Incidence/Risk Factors

It was recently recognized that lung cancer cells can evade immune surveillance by attenuating T-cell-mediated immune response among the tumor infiltrating lymphocytes (TILs) and by inducing anergy of regulatory (T-regs) and other T-cells that inhabit lung parenchyma and local lymph nodes. This prompted the development of several immune-checkpoint inhibitors (ICIs) that reactivate interactions between T-cells and lung cancer cells that revolutionized the treatment of lung and many other cancers [55–58]. Currently approved ICIs in front-line and/or subsequent treatment lines for lung cancer include pembrolizumab, nivolumab, and cemiplimab that target programmed cell death-1 (PD-1); atezolizumab and durvalumab that target programmed cell death-1-ligand 1 (PD-L1); and ipilimumab and tremelimumab that target cytotoxic T-lymphocyte antigen 4 (CTLA-4) [59]. Since the same mechanism of blocking PD-1, PD-L1 or CTLA-4 receptors to reinvigorate the T-cells and promote their antineoplastic cytotoxic activity is not specific to TILs, it can also lead to impaired immune tolerance in lung and other tissues, causing off-target inflammatory reactions named immune-related adverse effects (irAEs). ICI-P is a relatively uncommon but well-recognized and potentially life-threatening complication of ICI-based therapy for lung cancer [1, 60].

The incidence of any grade ICI-P in large phase 3 studies has been reported to be 1–7% (Table 4). Incidence of grade 3 or higher ICI-P has been reported in a meta-analysis by our group and others to be 0.5–3% [61] (Table 4). Several retrospective real-world studies have reported a higher incidence of ICI-P than what has been observed in clinical trials (Table 5). For example, in a recently published study that included 419 patients with NSCLC treated with ICIs, the cumulative incidence of ICI-P was found to be 9.5% and the main identified risk factor was ILD in never-smokers [62]. In another study of 315 patients with NSCLC, the incidence of ICI-P was also 9.5% and ICI-P-related mortality was 27%, with a median time to diagnosis of 52.5 days. Similarly, the presence of baseline lung fibrosis was significantly associated with the risk of development of ICI-P [63]. Other studies have shown ICI-P to develop in 14.6% of the patients with a median time to onset of 60 days (6–634 days); lung fibrosis score >  = 1 (on a scale 0–5) was the only variable associated with development of ICI-P [64]. A significantly higher incidence of ICI-P in patients with pre-existing ILD has been reported. An odds ratio of 6 was seen in one trial and a rate of 29% vs. 10% in another [9, 65]. It has also been noted that treatment-naïve patients tend to have higher rates of treatment-related pneumonitis [61].

**Table 4:** Incidence of ICI-P in pivotal phase 3 clinical trials investigating ICIs in lung cancer treatment

Agent Target	Histology / Stage	Clinical trial	Arms	Arm 1			Arm 2			Arm 3		
				Any grade	Grade 3 or higher	Grade 5	Any grade	Grade 3 or higher	Grade 5	Any grade	Grade 3 or higher	Grade 5
Pembrolizumab PD-1		Front-line										
	Stage IIIB or IV NSQ NSCLC	**KEYNOTE-021**	Pembrolizumab with carboplatin and pemetrexed vs. carboplatin and pemetrexed	4(7%)	not reported	not reported	0					
	Stage IV NSQ NSCLC	**KEYNOTE-189**	Pembrolizumab and carboplatin or cisplatin and pemetrexed vs. chemotherapy alone	13(4.4%)	9(3.1%)		4(2.8%)	3(2.1%)				
	Stage IV SQ or NSQ NSCLC	**KEYNOTE-024**	Pembrolizumab monotherapy vs. platinum-based chemotherapy in PD-L1 TPS > = 50%	9(5.8%)	4(2.6%)		1(0.7%)	1(0.7%)				
	Stage IV SQ NSCLC	**KEYNOTE-407**	Pembrolizumab with carboplatin and paclitaxel or albumin bound paclitaxel vs. chemotherapy alone	11(6.5%)	4(2.4%)		3(1.8%)	1(0.6%)				
	Stage IV SQ or NSQ NSCLC	**KEYNOTE-042**	Pembrolizumab vs chemotherapy in PD-L1 TPS > = 1%									
		Subsequent lines										
	Stage IIIB or IV SQ or NSQ NSCLC	**KEYNOTE-010**	Pembrolizumab vs. docetaxel	40(5.9%)	18(2.6%)		6(1.9%)	2(0.6%)				
	ES-SCLC	**KEYNOTE-028 & KEYNOTE-158 (pooled data)**	Pembrolizumab alone in patients with previous > = 1 line of therapy vs. > = 2 lines of therapy	2(1.5%)	1(0.8%)		1(1.2%)	1(1.2%)				
Atezolizumab PD-L1		Front-line										
	Adjuvant IB-IIIA NSCLC	**IMpower 010**	Atezolizumab vs. best supportive care following resection and platinum-based chemotherapy	19(4%)	4(< 1%)	1(< 1%)	3(< 1%)	0	0			
	Stage IV NSQ NSCLC	**IMpower 150**	Atezolizumab, bevacizumab, carboplatin and paclitaxel vs. bevacizumab, carboplatin and paclitaxel vs. atezolizumab, carboplatin, paclitaxel vs	9(2.3%)	4(1%)	0	0					
	ES-SCLC	**IMpower 133**	Atezolizumab with carboplatin and etoposide vs. carboplatin and etoposide	3(1.5%)	1(0.5%)	0	4(2%)	2(1%)				
		Subsequent lines										
	Stage IIIB/IV SQ or NSQ NSCLC	**OAK**	Atezolizumab vs. docetaxel	6(1%)	4(< 1%)		0					
Durvalumab PD-L1		Front-line										
	ES-SCLC	**CASPIAN**	Durvalumab plus platinum–etoposide vs. durvalumab plus tremelimumab plus platinum–etoposide vs. or platinum–etoposide alone	3(1.1%)		0	5(1.9%)		2(0.8%)	3(1.1%)		2(0.8%)
		Subsequent line (consolidation)										
	Stage III SQ or NSQ NSCLC	**PACIFIC**	Consolidation durvalumab vs. placebo following definitive chemoradiation therapy	43(9.1%)	6(1.3%)	4(0.8%)	8(3.4%)	2(0.9%)	2(0.9%)			
Ipilimumab/Nivolumab CTLA-4 / PD-1		Front-line										
	Neoadjuvant IB-IIIA NSCLC	**CheckMate 816**	Nivolumab plus platinum-based chemotherapy or platinum-based chemotherapy alone, followed by resection	2(1.1%)	0		1(0.6%)	1(0.6%)				
	Stage IV SQ or NSQ NSCLC	**CheckMate 227**	Nivo + ipi vs nivo alone vs chemotherapy			4 (< 1%)						1(< 1%)
	Stage IV SQ or NSQ NSCLC	**CheckMate 9LA**	Nivo + ipi + chemotherapy vs chemotherapy			1 (< 1%)			0			
		Subsequent lines										
	Stage IIIB/IV NSQ NSCLC	**CheckMate 057**	Nivolumab vs. docetaxel	4(1%)	3(1%)		0					
	Stage IIIB/IV SQ NSCLC	**CheckMate 017**	Nivolumab vs. docetaxel	2(2%)	1(1%)		0					
	ES- or LS-SCLC	**CheckMate 032**	Nivolumab alone (following 2 or more chemotherapy regimens)	2(1.8%)								
	ES- or LS-SCLC	**CheckMate 331**	Nivolumab vs. chemotherapy (amrubicin or topotecan)									
Cemiplimab PD-1		Front line										
	Stage IV SQ or NSQ NSCLC	**EMPOWER-Lung 1**	Cemiplimab vs. platinum-doublet chemotherapy	10(3%)	1(< 1%)	0	1(< 1%)	0	0			
	Stage III/IV SQ or NSQ NSCLC	**EMPOWER-Lung 3**	Cemiplimab with platinum-doublet chemotherapy vs. chemotherapy alone	13(4.2%)	1(0.3%)		1(0.7%)	0				
Tremelimumab CTLA-4		Front line										
	Stage IV NSCLC	**POSEIDON**	Tremelimumab, durvalumab, and platinum-based chemotherapy vs. durvalumab, and platinum-based chemotherapy vs. platinum-based chemotherapy	12(3.6%)	3(0.9%)		10(3%)	4(1.2%)		2(0.6%)	2(0.6%)	

SQ = squamous; NSQ = non-squamous; NSCLC = non-small cell lung *cancer*; ES-SCLC: extensive stage small-cell lung *cancer*; LS-SCLC: limited stage small-cell lung *cancer*

**Table 5:** Incidence of ICI-P in retrospective real-world studies

Study	Number of patients (N)	Incidence of any-grade ICI-P	Time to ICI-P onset	Treatment
Atchley et al. 2021	315	9.5%	52.5 days (IQR 21–128 days)	nivolumab (76.5%), pembrolizumab (22%)
Suresh et al. 2018	205	19%	82 days (IQR 20–183 days)	nivolumab (78%), pembrolizumab (11.2%), durvalumab (5.3%)
Altan et al. 2023	419	9.5%	215 days (IQR 120–330 days)	nivolumab (51.3%), atezolizumab (10.5%), durvalumab (4.7%), pembrolizumab (33.4%)
Fukihara et al. 2019	170	16%		nivolumab, pembrolizumab
Yamaguchi et al. 2018	123	14.6%	60 days (6–634 days)	nivolumab, pembrolizumab
Fujimoto et al. 2023	299	17.7%	123 days (78–159 days)	pembrolizumab with chemotherapy
Stuart et al. 2021	869	5.1%		ICI
Cho et al. 2018	167	13.2%		
Kanai et al. 2018	216	31% vs 12%	69 days (2–393 days)	nivolumab in patients with and without ILD
Shibaki et al. 2020	331	29% vs 10%	39–69 days (9–438 days)	anti-PD-1 in patients with and without ILD

Another single-center study reported that 16% of patients with NSCLC treated with single-agent nivolumab or pembrolizumab developed ICI-P. While 22 of those 27 patients recovered from ICI-P, the overall survival in those subjects was 8.7 months compared to 23 months in those who did not develop ICI-P. Those patients chose not to receive next-line NSCLC-directed therapy but rather best supportive care instead [66]. A plausible reason behind seeing more cases of ICI-P in clinic is due to the exclusion of individuals with ILD, radiation-induced pneumonitis and other preexisting fibrosing lung conditions from clinical trials participation, which appear to be major risk factors for the development of ICI-P.

The type of ICI agent used may be another risk factor for development of ICI-P. A meta-analysis of 19 trials reported that anti-PD-1 in comparison to anti-PD-L1 agents tend to be more frequently associated with ICI-P [61]. The main reason may be that anti-PD-1 (as opposed to anti-PD-L1) agents can block binding of PD-1 to both PD-L1 and PD-L2 receptors, which has been postulated to result in more pronounced disinhibition of T-cells [67]. In addition, incidence of treatment-related pulmonary toxicity between ICI in combination with chemotherapy and ICI alone or with other agents, was also reported by analyzing large worldwide VigiBase (World Health Organization's global Individual Case Safety Report database) and FAERS (FDA Adverse Event Reporting System) databases. The study found that anti-PD-L1/chemotherapy combination is not associated with significantly higher incidence of ICI-P, while anti-PD-1 and anti-CTLA4 chemotherapy combination was [68]. These findings can help inform decision-making when choosing between single-agent ICI and ICI-chemotherapy combination regimens, where applicable (e.g. tumor PD-L1 >  = 50%).

There may be distinct clinicopathologic differences between early (within the first 6 months) and late (after 6 months) onset ICI-P. The majority of cases were diagnosed within 2–3 months of ICI initiation and were likely to be more severe (grade > 2) and carry higher mortality [69].

### Management

In our current clinical practice, we utilize a multi-disciplinary approach in management of irAEs that includes a medical and radiation oncologist, radiologist, pulmonologist, pharmacist, palliative care practitioner, oncology nursing specialist, and social worker. The most challenging cases are presented to a specialized tumor board focused on irAEs. For grade 1 ICI-P, we typically hold therapy and re-evaluate with chest CT in 3 weeks. Patients who have no improvement or worsening findings on imaging despite holding therapy are treated as grade 2 pneumonitis. Grade 2 ICI-P is managed by holding therapy and initiation of corticosteroids, typically 1 mg/kg/day of prednisone, with close follow-up via phone or virtual visit within 2–3 days, followed by an in-person visit in 7 days. In absence of clinical deterioration, we taper prednisone by 10 mg/week over the following 4–6 weeks and recommend re-imaging with chest CT in 3–4 weeks. For grade 3 lung toxicity or worsening symptoms despite oral corticosteroids for grade 2 ICI-P, we advise hospitalization for close monitoring, detailed diagnostic work-up that may include bronchoscopy with bronchoalveolar fluid sampling for cytology and infectious etiology with or without lung biopsy, pulmonology consultation, and administration of 1–2 mg/kg/day of IV methylprednisolone. If this does not result in prompt improvement in clinical status within 48 h, we continue methylprednisolone or transition to 1 mg/kg/day of oral prednisone and add one of the following immunosuppressants and immunomodulators: mycophenolate mofetil, immunoglobulins (IVIG), infliximab, or cyclophosphamide. There are no specific clinical guidelines as to which agent to choose in such situations, and the efficacy/safety data for the above treatments is mixed. Naidoo and colleagues originally described the use of infliximab or infliximab with cyclophosphamide in five patients with progressive ICI-P despite corticosteroid treatment [10]. All five patients died, with three deaths from sepsis, one death from disease progression, and one death from pneumonitis. Since then, steroid refractory pneumonitis has been further defined as either failure to improve after at least 48 h of steroid treatment [64] or the addition of a second line agent after failure to improve or worsening of pneumonitis [65]. These two retrospective studies describing steroid-refractory pneumonitis found that mortality attributable to ICI-P and/or associated infectious complications was 23% and 75% [70]. In one study, patients treated with infliximab with or without IVIG had 100% mortality (5/5 patients) but those treated with IVIG alone had mortality of 42.9% (3/7 patients) [70]. In another study that evaluated steroid-refractory and steroid-resistant ICI-P, the rate of durable pneumonitis improvement with infliximab as the initial immunomodulator was 20% (4/20) and with mycophenolate it was 83% (5/6) [71]. However, it should be noted that only 2 patients in this cohort had grade 4 pneumonitis and both patients received infliximab alone, with one patient having no response and one patient having a transient response. None of the patients in the steroid refractory group received mycophenolate as the initial immunomodulator. Two other patients in the steroid-refractory group received mycophenolate after a transient response to infliximab; both patients had a durable response. A separate retrospective study examined 94 patients with ICI-P, with 9 patients (11%) receiving infliximab for grade 3 or 4 ICI-P. Four patients (44%) survived with sustained improvement, while five patients (56%) died from either progression of malignancy or multiorgan failure; two of the patients that died had initial improvement [72]. None of the published data available has included a substantial cohort of lung cancer patients.

The overall results of these retrospective studies are difficult to interpret. Many of the patients in these cohorts did not undergo bronchoscopy to reliably exclude infection. It seems plausible based on the response to IVIG that an overwhelming response to infection may drive the severe respiratory failure seen in this patient population. Therefore, infections should be aggressively sought out and treated prior to considering further immunosuppressive therapy. Once a decision is made to augment treatment, our approach has been to trial IVIG, infliximab, or mycophenolate mofetil. Due to high doses and anticipated prolonged course of corticosteroids in these instances, it is important to initiate supportive care including Pneumocystis jiroveci pneumonia (PJP), gastrointestinal (GI), and/or osteoporosis prophylaxis.

Rechallenge with ICIs after resolution of ICI-P remains controversial and requires an individualized approach, as it has been reported that patients with a history of ICI-P are at substantially higher risk (up to 50%) of re-developing ICI-P or another irAE [70]. Patients who recover from grade 4 ICI-P are not candidates for rechallenge with ICIs. Upon improvement or resolution of grade 3 lung toxicity, except in rare and carefully selected cases, re-treatment with ICIs is not recommended; rather consider continued close monitoring off therapy if a patient had favorable response or stable disease prior to development of toxicity, consider any available clinical trials (although likely to be limited by history of toxicity), or offer non-ICI next line of therapy. Upon resolution of grade 2 toxicity, re-initiation of ICI also requires careful consideration of any associated toxicities (concomitant irAEs, if any), other available treatment options, re-evaluation of ECOG performance status, and revisiting goals of care. If the patient experienced good clinical and/or radiographic response to ICI prior to development of toxicity, responds completely to corticosteroids alone without addition of a second agent, or has limited treatment options, our approach has been to offer re-initiation of the same ICI agent with or without low-dose prednisone, typically 20–30 mg/day. Patient follow-up via phone or virtually is then conducted in 2–3 days for any recurrence of pulmonary symptoms, then periodically, and subsequently patient is seen in clinic within 2–3 weeks with repeat chest imaging. If there is no radiographic recurrence of lung toxicity at the 3-week mark, prednisone is tapered over the following 1–2 weeks and then discontinued.

In terms of ICI-P chronicity, cases refractory to corticosteroid tapering over a period of >  = 12 weeks have been termed as chronic ICI-P by some authors [73, 74]. We rarely encounter such scenarios and management approaches have varied among treating oncologists and pulmonologists in the group. Generally, adding immunosuppressants similar to the above-described regimens is considered. While data on the use of these agents in chronic ICI-P is lacking, both mycophenolate mofetil and TNF-alpha inhibitors have been used successfully in the treatment of ILD associated with connective tissue disease, with a well-tolerated and predictable side effect profile [75]. It is the authors’ opinion that mycophenolate, 1.5-3gm daily divided in 2 doses, should be trialed first; with reservation of IVIG, 0.4gm/kg per dose given up to 5 doses, and infliximab, 5-10 mg/kg repeated every 2 weeks for 3 doses in cases where mycophenolate is either not tolerated or ineffective.

## Conclusion

Drug-induced pneumonitis in patients with lung cancer receiving systemic therapy is more common in real-world practice compared to what is reported in pivotal clinical trials. Most cases of pneumonitis are treatable with holding of the culprit drug and use of corticosteroids. Although relatively rare, it is important to have a high index of suspicion for more severe cases as they carry poor prognosis and high mortality; prompt recognition and treatment may improve outcomes. Since there are no universal guidelines for optimal escalation of treatment of severe cases of drug-induced pneumonitis, there is a need to test different treatment approaches of such patients in prospective studies.

## Key References

- Schneider, B.J., et al., Management of Immune-Related Adverse Events in Patients Treated With Immune Checkpoint Inhibitor Therapy: ASCO Guideline Update*.* J Clin Oncol, 2021. 39(36): p. 4073–4126.
  - This reference is of outstanding importance because it provides guidance for management of immune related adverse events as recommended by a panel of exeprts from different disciplines.
- Rugo, H.S., et al., Real-World Perspectives and Practices for Pneumonitis/Interstitial Lung Disease Associated With Trastuzumab Deruxtecan Use in Human Epidermal Growth Factor Receptor 2–Expressing Metastatic Breast Cancer*.* JCO Oncology Practice, 2023: p. OP.22.00480.
  - This reference is of importance because it provides guidance for management of penumonitis caused by ADC therapy as seen in practice rather than a clinical trial.
- Li, H., et al., Comparison of pneumonitis risk between immunotherapy alone and in combination with chemotherapy: an observational, retrospective pharmacovigilance study*.* Front Pharmacol, 2023. 14: p. 1142016.
  - This reference is of importance because it provides evidence that combination chemotherapy with immunotherapy leads to worse penumonitis especially with anti-PD-1 and anti-CTLA4 combination.

## Data Availability

No datasets were generated or analysed during the current study.
